# Compliance with Sport Injury Prevention Interventions in Randomised Controlled Trials: A Systematic Review

**DOI:** 10.1007/s40279-016-0470-8

**Published:** 2016-02-11

**Authors:** Miriam van Reijen, Ingrid Vriend, Willem van Mechelen, Caroline F. Finch, Evert A. Verhagen

**Affiliations:** 1 Department of Public & Occupational Health, EMGO+ Institute, VU University Medical Center, Amsterdam, The Netherlands; 2 Amsterdam Collaboration on Health & Safety in Sports, IOC Research Center, AMC/VUmc, Amsterdam, The Netherlands; 3 Consumer Safety Institute VeiligheidNL, Amsterdam, The Netherlands; 4 School of Human Movement and Nutrition Sciences, Faculty of Health and Behavioural Sciences, University of Queensland, Brisbane, Australia; 5 UCT/MRC Research Unit for Exercise Science and Sports Medicine (ESSM), Department of Human Biology, Faculty of Health Sciences, University of Cape Town, Cape Town, South Africa; 6 Australian Centre for Research into Injury in Sport and its Prevention (ACRISP), Federation University Australia, Ballarat, VIC Australia

## Abstract

**Introduction:**

Sport injury prevention studies vary in the way compliance with an intervention is defined, measured and adjusted for.

**Objective:**

The objective of this systematic review was to assess the extent to which sport injury prevention randomised controlled trials (RCTs) have defined, measured and adjusted results for compliance with an injury prevention intervention.

**Methods:**

An electronic search was performed in MEDLINE, PubMed, the Cochrane Center of Controlled Trials, CINAHL (Cumulative Index to Nursing and Allied Health Literature), PEDro (Physiotherapy Evidence Database) and SPORTDiscus. English RCTs, quasi-RCTs and cluster-RCTs were considered eligible. Trials that involved physically active individuals or examined the effects of an intervention aimed at the prevention of sport- or physical activity-related injuries were included.

**Results:**

Of the total of 100 studies included, 71.6 % mentioned compliance or a related term, 68.8 % provided details on compliance measurement and 51.4 % provided compliance data. Only 19.3 % analysed the effect of compliance rates on study outcomes. While studies used heterogeneous methods, pooled effects could not be presented.

**Conclusions:**

Studies that account for compliance demonstrated that compliance significant affects study outcomes. The way compliance is dealt with in preventions studies is subject to a large degree of heterogeneity. Valid and reliable tools to measure and report compliance are needed and should be matched to a uniform definition of compliance.

**Electronic supplementary material:**

The online version of this article (doi:10.1007/s40279-016-0470-8) contains supplementary material, which is available to authorized users.

## Key Points

**Table:** 

Compliance with injury prevention interventions can significantly affect study outcomes.
There is considerable heterogeneity in the way that sports injury prevention studies have measured, defined and reported compliance. More uniformity is needed in future studies to better progress sports injury prevention.

## Introduction

It is widely recognised that participation in regular sports and physical activity has the potential to improve health [1]. However, involvement in such activities also entails a risk of sustaining an injury. Serious sport injuries that take a considerable time to heal can force those involved not only to withdraw from the activity, but also to seek medical care and invest in medication and assisting materials—such as tape, braces and crutches. They can even prevent someone from continuing work or study activities. As a result, injuries lead not only to an individual burden, but also to substantial societal direct and indirect cost [2].

Numerous studies have been performed to evaluate the efficacy of interventions to prevent sport injuries or to reduce the risk of recurrent injury [3]. Although a variety of efficacious preventive interventions have been proposed, implementation of these interventions faces the challenge of persuading participants to follow instructions as prescribed. Establishing the effectiveness of any injury prevention intervention requires knowledge about what percentage of the targeted population exactly complied with the prescribed protocol. Especially in an intention-to-treat (ITT) approach, insights into the compliance to the intervention provides valuable and, arguably, necessary information to judge the efficacy of an intervention [4].

When one incorrectly assumes that the entire study population has complied with the intervention protocol, the preventive effect of any intervention can be either over- or underestimated. Unfortunately, many different definitions of compliance have been reported in the sports medicine literature [3]. Both the constructs of compliance and adherence have been used interchangeably to describe the complete and correct following of a prescribed intervention. Nonetheless, the two terms are not synonymous. Compliance refers to participant obedience in a study where a clinician/researcher prescribes the intervention, with little to no right of consultation on behalf of the participant. It can thus be defined as “the athletes’ correct following of the prescribed intervention” [4]. Adherence implicates a more collaborative environment in which a clinician/researcher and a study participant cooperate to develop an intervention that fits with the participants’ opportunities and restraints [5, 6]. Research, ideally performed in a more or less controlled setting, therefore implicitly focuses on compliance, rather than on adherence.

In addition to using correct definitions, the operationalisation of compliance requires attention. A comprehensive assessment of study results will only be possible if there is thorough insight into the way compliance has been defined, measured and adjusted for. If there is no, or incomplete, information available on the extent to which participants have complied with the intervention, it will remain unclear as to whether the intervention has been truly efficacious or not. Therefore, it is important that researchers, who aim to present studies of high quality with a low risk of bias, acknowledge the importance of compliance, and measure and report on compliance and its effects on study outcomes.

A number of study reporting guidelines, such as the STROBE (Strengthening the Reporting of Observational Studies in Epidemiology) statement and the CONSORT (CONsolidated Standards Of Reporting Trials) statement, recognise the importance of compliance and include specific items on the topic in their guidelines [7–9]. The STROBE statement addresses cohort, case-control and cross-sectional studies; the CONSORT statement specifically addresses the quality of reports of randomised controlled trials (RCTs).

Until 2010, the CONSORT statement advocated the use of ITT analysis for RCTs. ITT analysis does not include the measurement of compliance but assumes full adherence to the prescribed intervention [4]. However, as mentioned in the CONSORT statement, strict ITT analysis is often hard to achieve for two main reasons: missing outcomes for some participants and non-adherence to the protocol. Therefore, since 2010, the CONSORT statement has replaced the mention of ITT by the requirement of “more information on retaining participants in their original assigned groups” [7]. As an alternative to an ITT analysis, it has been suggested that per-protocol-analysis (PPA)—sometimes referred to as ‘modified ITT’—can be used [4]. In this approach, the analysis is performed only on those participants who have fully complied with the programme. A PPA can provide a measurement of efficacy in that it gives the result of a prescribed programme that is implemented exactly as the researcher originally developed it. It is currently unclear to what extent RCTs on sport injury prevention have included the guidelines provided by the CONSORT statement and to what extent compliance measures have been addressed. This systematic review therefore aims to assess the extent to which sport injury prevention RCTs have defined, measured and adjusted their results for compliance with the trialled intervention(s).

## Methods

### Research Questions

This review answers the following questions to provide a detailed analysis on how compliance has been reported in sport injury prevention studies:

1. How and how often was compliance *defined*?
2. When defined, how was compliance *measured*?
3. When defined and measured, how was the outcome *adjusted* for compliance in the analysis?

### Electronic Searches

Seven electronic databases were systematically searched for peer-reviewed publications on sport injury prevention interventions: PubMed (to October 2014), MEDLINE (1966 to October 2014), SPORTDiscus (1949 to October 2014), the Cochrane Central Register of Controlled Trials (to October 2014), CINAHL (Cumulative Index to Nursing and Allied Health Literature; 1982 to October 2014), PEDro (The Physiotherapy Evidence Database; to October 2014) and Web of Science (to October 2014). A standardised search strategy, based on a word string, including relevant sports injury terms and study designs, was used. The following keywords, and various combinations of those words, were used in the search: sport injury/ies, athletic injury/ies, prevention, preventive, preventi*, randomiz/s/ed, randomiz/s/ed controlled trial. Reference lists and related citations of included studies and relevant systematic reviews were also hand-searched for applicable publications.

#### Inclusion Criteria

Only RCTs, quasi-RCTs and cluster-RCTs were considered eligible for inclusion. The reason for including only (cluster- and/or quasi-)RCTs is that these studies maximise internal validity, which can be seen as a prerequisite for external validity. Trials were included that involved physically active individuals of either sex and of all ages. To be selected, studies had to examine the effects of an intervention aimed at the prevention of sport- or physical activity-related injuries. The primary outcome of the studies had to be a measure of sports- or physical activity-related injury (i.e. injury rate, time to first injury or the number of injured individuals). Only English-language publications were considered.

#### Exclusion Criteria

Studies that did not assess prevention of sports injury, that were not an RCT, quasi-RCT or cluster-RCT, or did not involve a physically active population were excluded from this review.

### Definitions

Compliance in this review was defined as “the athletes’ correct following of a prescribed intervention” [4]. It is acknowledged that a number of terms have been used in the scientific literature, referring to comparable constructs. As such, for the purpose of this current review, we considered all text referrals to participants’ following of an intervention as compliance. Other examples of phrases equivalent to compliance commonly used in publications are ‘use’, ‘cooperation’ and ‘adoption’ [4]. In this review, all studies included were scrutinised thoroughly to identify the specific form/phrase used by the authors. This ensured that all accounts of compliance were included.

### Methodological Quality

Potentially eligible studies were initially screened by title and abstract by the primary author. When eligibility was unclear, full-text articles were retrieved. In order to assess the methodological quality and risk of bias, all included studies were assessed based on ten out of 12 criteria as recommended by Furlan et al. [10]. These were the method of randomisation, concealed allocation, blinding of participants, blinding of care providers, blinding of outcome assessors, dropout rate, analysis according to allocated group, baseline similarity of the groups, compliance and timing of outcome assessment. This was done to assess if there were differences in the risk of bias between studies that did and did not report compliance. It is possible that studies that did not report compliance also failed to report other important methodological and design properties. Two criteria were omitted from Furlan et al. [10]—the reporting without selective outcome and avoidance of co-interventions—as these criteria were not considered to be distinctive for risk of bias between the included studies.

Each criterion was scored as ‘yes’, ‘unclear’ or ‘no’. Furlan et al. [10] defined studies with more than 6 points (yes = 1 point) as having “low risk of bias”. As two criteria were omitted, the original scoring was adjusted. Hence, more than 5 points was considered as the cut-off for “low risk of bias”.

To familiarize the authors with the risk of bias assessment, three reviewers (MvR, IV and EAV) scored ten studies that were randomly selected from all studies. Examining the disagreement in the assessment of these ten studies allowed the reviewers to identify possible incongruities in scoring. Thereafter, the total number of studies (*n* = 110) was randomly divided in two equal-sized sets (*n* = 55) and two reviewers (MvR and IV) both independently assessed risk of bias for one set. For the coding reliability assessment, from each of the sets, 19 studies were randomly selected. Both reviewers scored these 38 studies. It was agreed that if the agreement (kappa) score for these 38 studies was >0.9, agreement was acceptable and there was no need for the reviewers to score all studies separately. Of the 380 items that were scored twice, there was agreement on 370 items. This resulted in an agreement (kappa) score of 0.95. Based on this high level of agreement, it was thus decided that the remainder of the manuscripts did not needed to be assessed by both reviewers.

### Data Extraction

One reviewer (MvR) scrutinised the included studies for all terms referring to compliance. Thereafter, for the studies that mentioned compliance, details about the definitions, the methods of compliance measurements and the corresponding outcomes were extracted. Finally, all studies were examined for adjustment of the main outcome in their analyses by compliance rates.

## Results

### Search Results

**Fig. 1:**
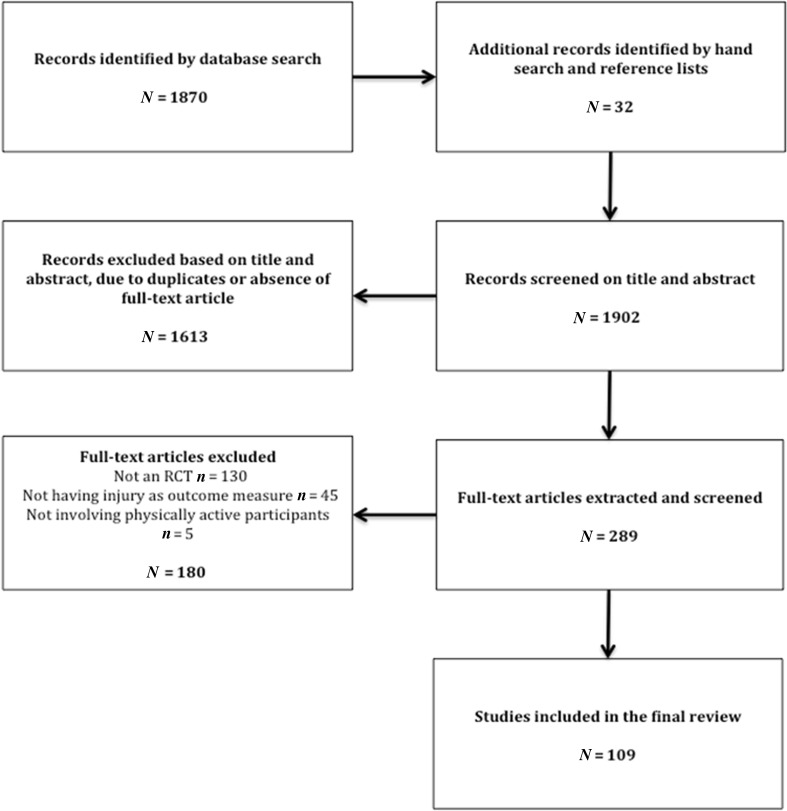
Literature search flow chart. *RCT* randomised controlled trial

**Fig. 2:**
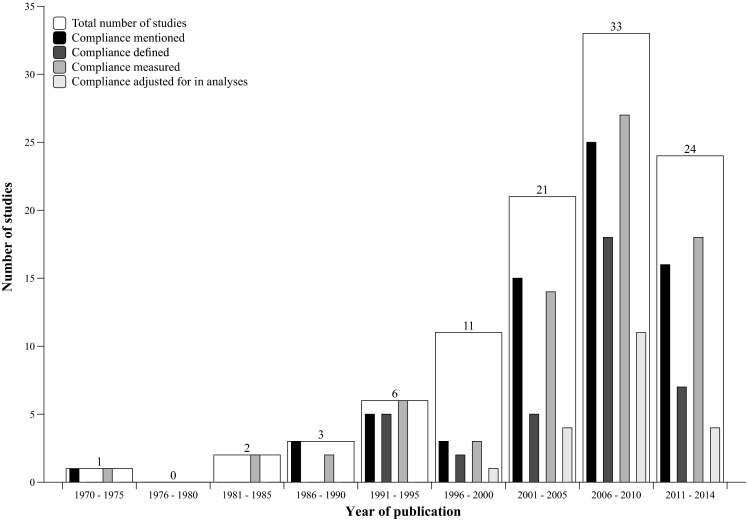
Annual trends in compliance reporting. *Note* A study can be categorised into more than one of the four categories shown

The search strategy initially yielded 1902 studies, of which a total of 289 full-text articles were retained after initial screening for eligibility. A total of 180 studies were then excluded (Fig. 1), resulting in 109 studies being included in this review. The primary reasons for exclusion were that studies did not involve an RCT or did not use injury as an outcome measure. For five studies, full-text articles could not be retrieved [11–15]. Electronic Supplementary Material Appendix S1 provides an overview of the studies included in the final review. Figure 2 describes the included studies in terms of their mentioning of, measurement of and/or adjustment for compliance.

### Risk of Bias Scores

The 109 included studies scored an average of 4.1 ± 1.8 yes ratings (out of 10), 2.8 ± 1.3 no ratings and 3.3 ± 1.8 unknown on the risk of bias assessment instrument. It can thus be concluded that, in general, the included studies demonstrated a fairly high ‘risk of bias’. The 21 studies that explicitly adjusted for compliance rates in their study outcomes—and hence had provided the most details on compliance in their report—scored an average of 4.7 ± 1.6 on the risk of bias assessment, compared with average scores of 3.9 ± 1.8 for the 88 studies that did not account for compliance. This suggests that the studies that accounted for compliance had a slightly higher methodological quality than those studies without such adjustment. Electronic Supplementary Material Appendix S1, Sect. 1 provides an overview of the risk of bias score of each of the included studies.

### Compliance

#### Terms Used for Compliance

Of all studies, 78 (71.6 %) mentioned compliance or a related term. Most common was the use of the term ‘compliance’ (*n* = 57; 52.3 %). Other terms used were ‘use’ (*n* = 8), ‘adherence’ (*n* = 6), ‘attendance’ (*n* = 2), ‘cooperation’ (*n* = 1) and ‘participation’ (*n* = 1). Some studies used multiple terms by switching between ‘compliance’ and ‘adherence’ (*n* = 2), ‘compliance’ and ‘exposure’ (*n* = 1) or ‘compliance’ and ‘internal dropout’ (*n* = 1). Electronic Supplementary Material Appendix S1, Sect. 2 provides an overview of the terms used in the included studies.

#### Measurements of Compliance

The majority of the 78 studies that mentioned compliance (75; 68.8 % of all studies included) provided details on how they measured compliance. Compliance rates were recorded using diverse methods. Studies that concerned supervised exercises derived compliance rates from a written or online report by a supervisor, e.g. a trainer, coach or designated team member (*n* = 15) [16–30]. Home-based or individual exercises studies made use of a written or online self-report (*n* = 12) [31–42]. In studies relating to the use of protective equipment (orthoses, wrist protectors, etc.) or supplements, this use was recorded by either the participant (*n* = 4) [43–46] or a supervisor (*n* = 5) [47–51]. In 15 studies [47, 52–65] the wearing/usage of protective equipment was only checked visually. In three studies [52, 54, 62], a lack of compliance with wearing/usage of material resulted in prohibition to participate; these studies therefore suggested 100 % compliance for people who remained in the study. For example, the participants who were designated to wear a helmet during football were visually checked before they entered the field; non-compliance with wearing the helmet resulted in the prohibition to play [52].

In 24 studies, researchers verified the reported compliance rates by multiple methods. These included combining self-report with the report of a supervisor [66–70], combining a report of a supervisor with random visits [5, 71–78], combining a report of a supervisor with phone calls and visits [79–81], combining self-report with random visits [82], combining a report of a supervisor with phone calls and emails [83] or combining self-report with phone calls [71].

Thirty-one studies included in this review were conducted in a military setting. Although it might be expected that a military setting would make it easier to report on compliance—with many supervised activities and a highly compliant environment—these studies did not provide more details on compliance than other studies. Slightly less than half of the military studies (*n* = 14) provided details on compliance measurements. In eight of these 14 studies it was reported that the participants were visually checked or supervised while carrying out the intervention. Two of those eight studies provided no further details on compliance rates [53, 54], two studies excluded participants from the analysis when they did not comply [55, 61] and the other four studies reported compliance rates of between 57 and 100 % [47, 56, 57, 60]. Electronic Supplementary Material Appendix S1, Sect. 3 provides an overview of ways in which studies have reported compliance rates.

#### Compliance Data and Adjustments for Compliance Rates

Of the 75 studies that provided information on compliance measurement, only 56 studies (51.4 % of all included studies) provided compliance data. These data were presented in heterogeneous ways. Nine studies [5, 16, 67, 71, 74, 79, 81, 84, 85] created subclasses of participants in which high, intermediate and low rates of compliance were defined. However, the (arbitrary) cut-off percentage that was considered for high versus low compliance varied considerably between studies.

For example, in a cluster-RCT on the FIFA 11+ injury prevention programme, low, middle and high compliance were defined respectively as performing <24.7, 24.8–48.1 or >48.2 % of all exercises [84]. This resulted in the categorization of 18 % of teams in the low compliance category, 41 % of teams within the moderate compliance category and 41 % of teams in the high category. In another neuromuscular training intervention cluster-RCT, high compliance was defined as carrying out three (of three) sessions in a first intensive intervention period, two sessions in the second intervention period and one session in the third/maintenance period [16]. In this study, 36 % of the teams were considered as highly compliant, 43 % as irregularly compliant and 21 % as having interrupted compliance.

Other studies choose to report compliance for each player [5, 73, 75, 78, 79, 81, 84], for the team as a whole [17–20, 72, 74, 75, 78, 79, 81] or a seasonal compliance rate [20, 79]. In addition, some studies combined compliance rates of the intervention and the control group, which were presented as one overall compliance rate [21, 22, 57, 66, 70, 82, 86]. Electronic Supplementary Material Appendix S1, Sect. 4 provides an overview of the studies that reported compliance data.

In addition to providing compliance rates, a mere 21 studies [5, 16, 17, 20, 23, 31, 32, 43, 58, 67, 71, 74, 76, 77, 79, 83–87] (19.3 % of all included studies) analysed the effect of different compliance rates on study outcomes. As the studies used heterogeneous methods to report these analyses, it is impossible to provide a pooled effect of compliance rates. Therefore, Table 1 presents the details of the effect of measured compliance rates on their study outcome in these 21 studies.

**Table 1:** Studies that analyse the effect of compliance rates on study outcome

Study (year)	Intervention	Population (intervention [*n*]/control [*n*]; % male)	Reported compliance rate in groups being compared	Analysis of the effect of compliance on study outcome
Cobb et al. [43] (2007)	Oral contraceptives	Young distance runners (69/81; 0)	74.5 % were seen as compliant. Compliance was defined as using >6 months of oral contraceptives	Compliant women were significantly protected against fractures (by 77 %), though this estimate was weakened when we excluded fractures that occurred early in the trial (58 % reduction in risk, *P* = 0.20). The researchers do mention that this finding could have been the result of chance or bias as it was found that women who switched from the control group to oral contraceptive use were less likely to have a history of stress fractures before joining the study
Emery et al. [71] (2005)	Home-based balance training	PE students (66/61; 50)	No report of specific compliance rates	Effect of compliance on static but not dynamic balance. Compliance with balance training sessions had an effect on the change in static balance: the observed change among students in the intervention group who reported fewer than 18 sessions over 6 weeks was holding their balance for 6.1 s (95 % CI –8.4–20.7), as compared with 25.8 s (95 % CI 16.4–35.1) among those who reported 18 or more sessions. Compliance did not have a significant effect on change in dynamic balance
Engebretsen et al. [31] (2008)	Exercise programme	Soccer players (315/193; 100)	Compliance was defined as completing >30 sessions: 29.2 % for knee exercises, 21.1 % for hamstring and 19.4 % for groin exercises	No difference was detected in the risk of knee injury between players in the high-risk intervention group who were compliant with the knee programme (0.2 [95 % CI −0.2 to 0.7] injuries per 1000 h) and the high-risk players in the high-risk control group (0.5 [95 % CI 0.2–0.9] injuries per 1000 h; RR = 0.46; 95 % CI 0.1–3.7). In the same way, no difference was observed in the incidence of hamstring (RR = 0.94; 95 % CI 0.3–3.2) and groin injuries (RR = 1.6; 95 % CI 0.5–5.6) between players in the high intervention group who were compliant with the respective training programmes and the high control group
Gabbe et al. [23] (2006)	Eccentric hamstring exercises	Amateur Australian Football players (114/106; 100)	46.8 % participated in >2 sessions	Significant difference due to compliance. When only control and intervention group players who participated in at least the first 2 sessions were analysed, a trend towards a protective effect for the intervention group was noticed (RR 0.3, 95 % CI 0.1–1.4; *P* = 0.098). Only 4 % of the compliant group sustained an injury vs. 13.2 % in the control group (no *P* value specified)
Hagglund et al. [74] (2013)	Neuromuscular training programme	Soccer players (2471/2085; 0)	79 % team compliance. Team compliance was defined as completing a supervised neuromuscular training	Teams with the highest level of compliance (89 %) had 88 % lower risk of re-injury rate than control and low compliance (63 %) teams. Low compliance and control were not significantly different
Hupperets et al. [32] (2009)	Proprioceptive training	Athletes with an ankle sprain (256/266; 52.4)	23 % fully compliant, 29 % partially compliant, 35 % non-compliant, 13 % unknown. A definition of compliance was not provided	Although a significant reduction in risk of injury was found in all groups, the researchers suggest that a higher compliance might have resulted in fewer recurrent injuries
Kiani et al. [20] (2010)	Exercise programme + education	Soccer players (777/729; 0)	6 % of players were 50 % compliant, 75 % were 75 % compliant and 18 % were 100 % compliant. A definition of compliance was not provided	Including only compliant teams: there was a non-significant different rate ratio in the intervention group compared with controls for all injuries (0.17 [95 % CI 0.02–0.75]) and for non-contact injury (0.11 [95 % CI 0.02–0.77])
Larsen et al. [68] (2002)	Custom-made shoe orthoses	Conscripts (77/69; 99.3)	88.3 % overall (control and intervention). A definition of compliance was not provided	ITT analysis gave an RR of 0.7 (95 % CI 0.5–1.1) and PPA analysis gave an RR of 0.3 (95 % CI 0.1–0.7)
Longo et al. [83] (2012)	FIFA 11+ warm-up	Basketball players (80/41; 100)	100 % compliance. A definition of compliance was not provided	In the intervention group, IIDs were lower than those in the control group for overall injuries (0.95 vs. 2.16; *P* = 0.0004), training injuries (0.14 vs. 0.76; *P* = 0.007), lower-extremity injuries (0.68 vs. 1.4; *P* = 0.022), acute injuries (0.61 vs. 1.91; *P* = 0.0001) and severe injuries (0 vs. 0.51; *P* = 0.004). The intervention group also had lower injury rates for trunk (0.07 vs. 0.51; *P* = 0.013), leg (0 vs. 0.38; *P* = 0.007), and hip and groin (0 vs. 0.25; *P* = 0.023) than in the control group. Differences in match injuries, knee injuries, ankle injuries and overuse injuries between 2 groups were not significant
Machold et al. [58] (2002)	Wrist protectors	Students (342/379; 60)	96.5%. A definition of compliance was not provided	The risk of severe wrist injury decreased by a factor of 0.13 using the protector
McIntosh et al. [76] (2009)	Padded headgear	Rugby players (1493/1128/1474; 100)^a^	Standard: 48.9 %; modified: 40.1 %. Compliance was defined as wearing headgear	Head injury and concussion rates based on headgear-wearing compliance were not significantly different
Myklebust et al. [17] (2007)	Neuromuscular training	Handball players (850/942; 0)	First season 26 and 42 % elite, second season 29 and 50 % elite, youth 87 %. Compliance was defined as conducting a minimum of 15 ACL injury prevention sessions during the 5- to 7-week period with >75 % of the athletes participating	There was downward trend in the number of injuries during the study period, as compliance seemed to improve. Due to a crossover effect of 22 %, both teams showed a significant lower rate of injuries
Pasanen et al. [16] (2008)	Neuromuscular training	Floor ball players (256/201; 0)	74 %. High compliance was defined as carrying out at least 3 training sessions a week during the first intensive period, at least twice a week during the second intensive period, and at least once a week during the maintenance weeks	Intervention teams with high compliance to the neuromuscular training had a lower risk of injury than the control group: the incidence rate ratio between the high compliance group and control group for non-contact leg injury was 0.19 (95 % CI 0.06–0.64, *P* = 0.007), for non-contact ankle ligament injury was 0.19 (95 % CI 0.05–0.82, *P* = 0.026), and for non-contact knee ligament injury was 0.32 (95 % CI 0.04–2.59, *P* = 0.284)
Ronning et al. [87] (2001)	Wrist protectors	Snowboarders (2515/2514; 64.2)	99.5 %. A definition of compliance was not provided	In the braced group, 8 wrist injuries (3 fractures and 5 sprains) were recorded, compared with 29 wrist injuries (2 fractures and 27 sprains) recorded in the control group. Considering all types of injuries, a total of 33 injuries occurred in the braced group and 51 in the control group. This is a significant difference in favour of the braced group (Chi-square test = 3.9, *P* = 0.05)
Soderman et al. [67] (2000)	Balance board training	Soccer players (62/78; 0)	70 %. Compliance is defined as performing >70 sessions	In the intervention group, no significant difference was found in the number of traumatic injuries or injured players between the compliant (*n* = 27) and non-compliant (*n* = 35) subgroups
Soligard et al. [79] (2008)	FIFA 11+ warm-up	Football players (1055/1220; 0)	77 % (team) and 57.9 % (player). High compliance 33–95 sessions, intermediate compliance 15–32 sessions, low compliance 0–14 sessions	The risk of injury was 35 % lower in intervention players in the third with the highest compliance (2.6 [95 % CI 2.0–3.2] injuries/1000 player hours) than in players in the intermediate third (4.0 [95 % CI 3.0–5.0] injuries/1000 player hours) (rate ratio 0.65, 95 % CI 0.44–0.94, *P* = 0.02). The 32 % reduction in risk of injury compared with the third with the lowest compliance (3.7 [95 % CI 2.2–5.3] injuries/1000 player hours) did not reach significance (rate ratio 0.68, 95 % CI 0.41–1.12, *P* = 0.13)
Soligard et al. [81] (2010)	FIFA 11+ warm-up	Football players (1055/1220; 0)	77 % (team) and 57.9 % (player). High compliance 33–95 sessions, intermediate compliance 15–32 sessions, low compliance 0–14 sessions	The risk of injury was 35 % lower in intervention players in the third with the highest compliance (2.6 [95 % CI 2.0–3.2] injuries/1000 player hours) than in players in the intermediate third (4.0 [95 % CI 3.0–5.0] injuries/1000 player hours) (rate ratio 0.65, 95 % CI 0.44–0.94, *P* = 0.02). The 32 % reduction in risk of injury compared with the third with the lowest compliance (3.7 [95 % CI 2.2–5.3] injuries/1000 player hours) did not reach significance (rate ratio 0.68, 95 % CI 0.41–1.12, *P* = 0.13). Furthermore, the risk of an acute injury was 39 % (*P* = 0.008) lower for players in the tertile with the highest compliance than in players in the intermediate tertile, whereas a 35 % reduction of injury risk compared with the tertile with the lowest compliance was not statistically significant (*P* = 0.09)
Steffen et al. [80] (2008)	FIFA 11+ warm-up	Football players (1091/1001; 0)	52 %. Compliance >20 sessions, non-compliance >20 sessions	In a sub-group analysis to determine whether compliance with the intervention program could have influenced the risk for injuries throughout the study period, it was shown that there was no difference in the injury incidence of overall and acute injuries between the compliant group and the non-compliant group
Steffen et al. [84] (2013)	FIFA 11+ warm-up	Football players (129/121/135; 0)^b^	Intervention 1—high, medium, low compliance: 52, 23, 25 %. Intervention 2—high, medium, low compliance: 41, 41, 18 %. Team compliance was defined as the proportion of all possible sessions where the 11+ was delivered, the number of team 11+ sessions/week and the mean number of team 11+ exercises/session	The unadjusted overall injury rate for players categorised into the high compliance group was 57 % lower than the injury rate of players in the low adherence group (IRR = 0.43, 95 % CI 0.19–1.00). However, adjusting for the cluster, age group, level of play and injury history, this between-group difference in injury risk was not statistically significant (IRR = 0.44, 95 % CI 0.18–1.06). No other dose–response relationship between high and low adherence to the 11+ and injury risk could be identified
Steffen et al. [85] (2008)		Football players (1091/1001; 0)	During the first 4 months of the season, the training program was used during 60 % of the football training sessions. Only 24 % of the intervention teams completed more than 20 prevention training sessions and were seen as compliant	There were no significant differences in incidence of overall and acute injuries between the compliant and non-compliant groups
Walden et al. [77] (2012)	Neuromuscular training	Football players (2479/2080; 0)	52.5 %. Compliance defined as >1 session per week	An adjusted subgroup analysis of compliant players (1303 players in 112 intervention group clubs, 1967 players in 106 control group clubs) showed a statistically significant 83 % rate reduction in anterior cruciate ligament injury (rate ratio 0.17, 95 % CI 0.05–0.57, *P* = 0.004), as well as significant reductions for secondary outcomes in the intervention group compared with the control group (severe knee injury rate ratio 0.18, 95 % CI 0.07–0.45, *P* < 0.001; any acute knee injury rate ratio 0.53, 95 % CI 0.30–0.94, *P* = 0.03). Analyses of non-contact anterior cruciate ligament injuries showed a reduction in rates favouring the intervention group. The reduction was statistically significant only for the adjusted subgroup analysis of compliers (ITT analysis rate ratio 0.40, 95 % CI 0.13–1.18, *P* = 0.10; adjusted subgroup analysis rate ratio 0.26, 95 % CI 0.07–0.99, *P* = 0.049)

*ACL* anterior cruciate ligament, *IID* injury incidence density, *IRR* incidence rate ratio, *ITT* intention-to-treat, *PE* physical education, *PPA* per-protocol-analysis-treat, *RR* relative risk

^a^In this study, participants were assigned to 3 different study groups: the control group, the standard headgear group and the modified headgear group

^b^In this study, participants were assigned to 3 different study groups: an unsupervised group, a group who received coach-led workshops and a group who received coach-led workshops and on-field supervision

## Discussion

### A Lack of a Uniform Definition of Compliance

In the studies presented in this review, various methods were employed to define, measure and analyse the effect of compliance. The most important finding is that, although the majority of studies mention the concept of compliance, there is a large degree of heterogeneity in the manner in which studies deal with this concept. Some studies merely mention compliance in either the introduction or discussion without providing further details on compliance assessment and compliance data. As can be seen from Fig. 2, there are more studies that provide compliance data than there are studies that give an explicit definition of compliance or one of the related constructs. In other words, whilst many report compliance, a majority do not define this term or explicitly state how they operationalised it.

The majority of the studies report minimal details on (1) the definition of compliance; (2) how compliance was measured; (3) the frequency by which compliance was measured (every day, week, month); and (4) how compliance affected study outcomes.

From 1970 onwards there was a clear increase in the number of sport injury prevention RCT studies. However, in the last few years (2011–2014) this trend has not continued and the number of injury prevention RCTs has actually decreased. It is likely that after numerous efficacy studies, research now focuses on implementation of prevention measures in non-RCT studies. As these non-RCT studies are not the topic of this review, they will not appear in Fig. 2.

### The Importance of Compliance Reporting

In order to evaluate study outcomes in the context in which they are examined, it is essential that studies report the percentage of participants who have actually complied with the prescribed intervention. Compliance to an intervention significantly influences the outcomes of intervention studies, which is clearly illustrated by a number of studies included in this review [5, 23, 32, 71, 74]. For example, in the study by Steffen et al. [5] that assessed compliance rates to a neuromuscular injury prevention programme, high, intermediate and low compliance groups were defined. The authors’ PPA found that only the high compliant group benefited significantly from the intervention.

In the study by Emery et al. [71] evaluating home-based balance training, participants who had conducted more than 18 sessions (of the recommended 42 sessions) in 6 weeks had achieved a significant improvement in static balance skills. Participants with lower compliance rates did not improve their static balance skills. Gabbe et al. [23] evaluated eccentric hamstring exercises in amateur football players, of whom only 4 % of those who were compliant with the intervention sustained an injury. Players who were not compliant to the intervention showed no reduced injury risk when compared to the control group. Hagglund et al. [74] reported similar outcomes, showing that a significant reduction in injury rates was found only in teams with the highest compliance to a neuromuscular training programme. Finally, the study of Hupperets et al. [32], in which only 23 % of participants were fully compliant, suggested that higher compliance would have resulted in fewer injuries. In a secondary analysis in a subsequent paper, it was indeed shown that the small group of participants with high compliance was responsible for the positive effect of the exercise programme on recurrent injury risk [4].

Information on the rate of compliance and its effect on study outcomes can be shaped into a clear message for the target groups involved; they should be informed about the number of training sessions they should at least participate in to reduce their risk of sustaining an injury. Providing information on compliance rates and the effect of those different rates on study outcomes might increase the practical usability of study results for the target group.

### Acknowledgment of the CONSORT Statement

The CONSORT statement argues that, in order to evaluate both efficacy (with the assumption of full compliance and no recognition of implementation barriers) and effectiveness (the real-life adoption of an intervention), researchers should analyse study results using ITT, PPA and a graded compliance measure [7]. The latter refers to the extent to which participants have complied with the programme and what effect this has had on the outcome.

In addition to the diversity by which compliance is defined, measured and adjusted for in the analysis, the studies included in this review show a large degree of heterogeneity in the use of ITT, PPA or graded compliance.

Thirty-seven studies have used one or more of the recommended analyses. Twenty-eight studies [16, 17, 27, 29, 32, 34, 37–42, 44, 50, 52, 71, 72, 75–82, 84, 88, 89] used ITT analysis, one used PPA [19] and eight studies [23, 31, 43, 47, 58, 86, 90, 91] used both analyses (see Electronic Supplementary Material Appendix S1). It is clear that, although the CONSORT statement clearly acknowledges the importance of compliance and, hence, provides a step forward in improving the quality of intervention studies, there is still a lack of uniformity. What is needed is a uniform way in which compliance is dealt with.

### Further Research

Further research needs to confirm which measures provide the most valid and reliable assessment of compliance. Although various methods have been used to measure compliance (e.g. the use of written, vocal or online self-reports, supervision and/or unscheduled visits), each method has its own limitations. Participants can incorrectly recall their activities or provide socially desirable reports on self-reported measures of compliance. In addition, a uniform definition of compliance and a categorisation of compliance rates might increase the possibility of comparing the effectiveness of different injury prevention programmes. The main weakness of the current study is that it only included RCTs. It would be of interest to conduct a similar review that includes both RCTs and less-controlled studies to identify adherence to sport injury intervention studies in which the setting is less controlled.

## Conclusion

Injury prevention studies vary significantly in the way they define, measure and adjust for compliance. While the majority of these studies mention the concept of compliance, only one-fifth of the studies gave a more detailed account of how compliance rates influence their study results. The studies that did account for compliance demonstrate that the level of compliance can have a significant effect on study outcomes. Valid and reliable tools to measure and report compliance need to be developed, matched to a uniform definition of compliance. Although current guidelines for reporting of studies have increased awareness of the need for compliance measurements, the way these measurements are executed and reported still deals with a large degree of heterogeneity.

## Electronic supplementary material

Below is the link to the electronic supplementary material.

Supplementary material 1 (DOCX 52 kb)

## References

[1] Landry BW, Driscoll SW (2012). Physical activity in children and adolescents. PM R.

[2] Newnam S, Collie A, Vogel AP (2014). The impacts of injury at the individual, community and societal levels: a systematic meta-review. Public Health.

[3] Klugl M, Shrier I, McBain K (2010). The prevention of sport injury: an analysis of 12,000 published manuscripts. Clin J Sports Med.

[4] Verhagen EALM, Hupperets MDW, Finch CF (2011). The impact of adherence on sports injury prevention effect estimates in randomised controlled trials: Looking beyond the CONSORT statement. J Sci Med Sport..

[5] Steffen K, Emery CA, Romiti M (2013). High adherence to a neuromuscular injury prevention programme (FIFA 11+) improves functional balance and reduces injury risk in Canadian youth female football players: a cluster randomised trial. Br J Sports Med.

[6] Sabaté E (2003). Adherence to long-term therapies.

[7] Schulz KF, Altman DG, Moher D (2010). CONSORT 2010 statement: updated guidelines for reporting parallel group randomised trials. BMC Med.

[8] Yoon U, Knobloch K (2012). Quality of reporting in sports injury prevention abstracts according to the CONSORT and STROBE criteria: an analysis of the World Congress of Sports Injury Prevention in 2005 and 2008. Br J Sports Med.

[9] Sorensen AA, Wojahn RD, Manske MC (2013). Using the strengthening the reporting of observational studies in epidemiology statement (STROBE) to assess reporting of observational trials in hand surgery. J Hand Surg Am.

[10] Furlan AD, Pennick V, Bombardier C (2009). Editorial Board, Cochrane Back Review Group. 2009 updated method guidelines for systematic reviews in the Cochrane Back Review Group. Spine (Phila Pa 1976).

[11] Amaroso P, Ryan J, Bickley B (1998). Braced for impact: Reducing military paratroopers’ ankle sprains using outside-the-boot braces. J Trauma.

[12] Amako M, Oda T, Masuoka K (2003). Effect of static stretching on prevention of injuries for military recruits. Mil Med.

[13] Fauno P, Kalund S, Andreasen I (1993). Soreness in lower extremities and back is reduced by use of shock absorbing heel inserts. Int J Sports Med.

[14] Frey C, Feder KS, Sleight J (2010). Prophylactic ankle brace use in high school volleyball players: a prospective study. Foot Ankle Int.

[15] Bixler B, Jones RL (1992). High-school football injuries: effects of a post-halftime warm-up and stretching routine. Fam Pract Res J.

[16] Pasanen K, Parkkari J, Pasanen M (2008). Neuromuscular training and the risk of leg injuries in female floorball players: cluster randomised controlled study. Br J Sports Med.

[17] Myklebust G, Engebretsen L, Braekken IH (2007). Prevention of noncontact anterior cruciate ligament injuries in elite and adolescent female team handball athletes. Instr Course Lect.

[18] Brushoj C, Larsen K, Albrecht-Beste E (2008). Prevention of overuse injuries by a concurrent exercise program in subjects exposed to an increase in training load. Am J Sports Med.

[19] Gilchrist J, Mandelbaum BR, Melancon H (2008). A randomized controlled trial to prevent noncontact anterior cruciate ligament injury in female collegiate soccer players. Am J Sports Med.

[20] Kiani A, Hellquist E, Ahlqvist K (2010). Prevention of soccer-related knee injuries in teenaged girls. Arch Intern Med.

[21] Fredberg U, Bolvig L, Andersen NT (2008). Prophylactic training in asymptomatic soccer players with ultrasonographic abnormalities in Achilles and patellar tendons: the Danish Super League Study. Am J Sports Med.

[22] Coppack RJ, Etherington J, Wills AK (2011). The effects of exercise for the prevention of overuse anterior knee pain: a randomized controlled trial. Am J Sports Med.

[23] Gabbe BJ, Branson R, Bennell KL (2006). A pilot randomised controlled trial of eccentric exercise to prevent hamstring injuries in community-level Australian Football. J Sci Med Sport.

[24] Ekstrand J, Gillquist J, Liljedahl S-O (1983). Prevention of soccer injuries—supervision by doctor and physiotherapist. Am J Sports Med.

[25] Surve I, Schwellnus MP, Noakes T (1994). A fivefold reduction in the incidence of recurrent ankle sprains in soccer players using the Sport-Stirrup orthosis. Am J Sports Med.

[26] Verhagen E, van der Beek A, Twisk J (2004). The effect of a proprioceptive balance board training program for the prevention of ankle sprains: a prospective controlled trial. Am J Sports Med.

[27] Olsen O-E, Myklebust G, Engebretsen L (2005). Exercises to prevent lower limb injuries in youth sports: cluster randomised controlled trial. BMJ.

[28] McGuine TA, Keene JS (2006). The effect of a balance training program on the risk of ankle sprains in high school athletes. Am J Sports Med.

[29] Hagglund M, Walden M, Ekstrand J (2007). Lower reinjury rate with a coach-controlled rehabilitation program in amateur male soccer: a randomized controlled trial. Am J Sports Med.

[30] Petersen J, Thorborg K, Nielsen MB (2011). Preventive effect of eccentric training on acute hamstring injuries in men’s soccer a cluster-randomized controlled trial. Am J Sports Med.

[31] Engebretsen AH, Myklebust G, Holme I (2008). Prevention of injuries among male soccer players: a prospective, randomized intervention study targeting players with previous injuries or reduced function. Am J Sports Med.

[32] Hupperets MDW, Verhagen EALM, van Mechelen W (2009). Effect of unsupervised home based proprioceptive training on recurrences of ankle sprain: randomised controlled trial. BMJ..

[33] van Mechelen W, Hlobil H, Kemper HC (1993). Prevention of running injuries by warm-up, cool-down, and stretching exercises. Am J Sports Med.

[34] Wedderkopp N, Kaltoft M, Holm R (2003). Comparison of two intervention programmes in young female players in European handball–with and without ankle disc. Scand J Med Sci Sports.

[35] Sherry MA, Best TM (2004). A comparison of 2 rehabilitation programs in the treatment of acute hamstring strains. J Orthop Sports Phys Ther..

[36] Noh Y-E, Morris T, Andersen MB (2007). Psychological intervention programs for reduction of injury in ballet dancers. Res Sports Med.

[37] Buist I, Bredeweg SW, van Mechelen W (2008). No effect of a graded training program on the number of running-related injuries in novice runners a randomized controlled trial. Am J Sports Med.

[38] Cumps E, Verhagen E, Duerinck S (2008). Effect of a preventive intervention programme on the prevalence of anterior knee pain in volleyball players. Eur J Sport Sci.

[39] Jamtvedt G, Herbert RD, Flottorp S (2010). A pragmatic randomised trial of stretching before and after physical activity to prevent injury and soreness. Br J Sports Med.

[40] Parkkari J, Taanila H, Suni J (2011). Neuromuscular training with injury prevention counselling to decrease the risk of acute musculoskeletal injury in young men during military service: a population-based, randomised study. BMC Med.

[41] Bredeweg SW, Zijlstra S, Bessem B (2012). The effectiveness of a preconditioning programme on preventing running-related injuries in novice runners: a randomised controlled trial. Br J Sports Med.

[42] Janssen KW, van Mechelen W, Verhagen EALM (2014). Bracing superior to neuromuscular training for the prevention of self-reported recurrent ankle sprains: a three-arm randomised controlled trial. Br J Sports Med.

[43] Cobb KL, Bachrach LK, Sowers M (2007). The effect of oral contraceptives on bone mass and stress fractures in female runners. Med Sci Sports Exerc.

[44] Kekkonen RA, Vasankari TJ, Vuorimaa T (2007). The effect of probiotics on respiratory and gastrointestinal symptoms during training in marathon runners. Int J Sports Nutr Exerc Metab.

[45] Ryan MB, Valiant GA, McDonald K (2011). The effect of three different levels of footwear stability on pain outcomes in women runners: a randomised control trial. Br J Sports Med.

[46] Theisen D, Malisoux L, Genin J (2014). Influence of midsole hardness of standard cushioned shoes on running-related injury risk. Br J Sports Med..

[47] Milgrom C, Finestone A, Novack V (2004). The effect of prophylactic treatment with risedronate on stress fracture incidence among infantry recruits. Bone.

[48] Schwellnus MP, Jordaan G (1992). Does calcium supplementation prevent bone stress injuries? A clinical trial. Int J Sports Nutr.

[49] Kinchington MA, Ball KA, Naughton G (2011). Effects of footwear on comfort and injury in professional rugby league. J Sports Sci..

[50] McGuine TA, Brooks A, Hetzel S (2011). The effect of lace-up ankle braces on injury rates in high school basketball players. Am J Sports Med.

[51] McGuine TA, Hetzel S, Wilson J (2012). The effect of lace-up ankle braces on injury rates in high school football players. Am J Sports Med.

[52] Kraus JF, Anderson BD, Mueller CE (1970). An investigation of the effectiveness of a new helmet to control touch football head injuries. Am J Public Health Nations Health.

[53] Milgrom C, Finestone A, Shlamkovitch N (1992). Prevention of overuse injuries of the foot by improved shoe shock attenuation: a randomized prospective study. Clin Orthop Relat Res.

[54] Finestone A, Giladi M, Elad H (1999). Prevention of stress fractures using custom biomechanical shoe orthoses. Clin Orthop Relat Res.

[55] Knapik JJ, Swedler DI, Grier TL (2009). Injury reduction effectiveness of selecting running shoes based on plantar shape. J Strength Cond Res.

[56] Knapik J, Hauret G, Arnold S (2003). Injury and fitness outcomes during implementation of physical readiness training. Int J Sports Med.

[57] Finestone A, Novack V, Farfel A (2004). A prospective study of the effect of foot orthoses composition and fabrication on comfort and incidence of overuse injuries. Foot Ankle Int.

[58] Machold W, Kwasny O, Eisenhardt P (2002). Reduction of severe wrist injuries in snowboarding by an optimized wrist protection device: a prospective randomized trial. J Trauma.

[59] Milgrom C, Giladi M, Kashtan H (1985). A prospective study of the effect of a shock-absorbing orthotic device on incidence of stress fractures in military recruits. Foot Ankle Int.

[60] Sitler M, Ryan J, Hopkinson W (1990). The efficacy of a prophylactic knee brace to reduce knee injuries in football. A prospective, randomized study at West Point. Am J Sports Med.

[61] Barrett JR, Tanji JL, Drake C (1993). High-versus low-top shoes for the prevention of ankle sprains in basketball players. A prospective randomized study. Am J Sports Med.

[62] Sitler M, Ryan J, Wheeler B (1994). The efficacy of a semirigid ankle stabilizer to reduce acute ankle injuries in basketball a randomized clinical study at West Point. Am J Sports Med.

[63] Finch C, Braham R, McIntosh A (2005). Should football players wear custom fitted mouthguards? Results from a group randomised controlled trial. Inj Prev.

[64] Mickel TJ, Bottoni CR, Tsuji G (2006). Prophylactic bracing versus taping for the prevention of ankle sprains in high school athletes: a prospective, randomized trial. J Foot Ankle Surg.

[65] George SZ, Childs JD, Teyhen DS (2011). Brief psychosocial education, not core stabilization, reduced incidence of low back pain: results from the Prevention of Low Back Pain in the Military (POLM) cluster randomized trial. BMC Med.

[66] Emery CA, Meeuwisse WH (2010). The effectiveness of a neuromuscular prevention strategy to reduce injuries in youth soccer: a cluster-randomised controlled trial. Br J Sports Med.

[67] Soderman K, Werner S, Pietila T (2000). Balance board training: prevention of traumatic injuries of the lower extremities in female soccer players? A prospective randomized intervention study. Knee Surg Sports Traumatol Arthrosc.

[68] Larsen K, Weidick F, Leboeuf-Yde C (2002). Can passive prone extensions of the back prevent back problems?: A randomized, controlled intervention trial of 314 military conscripts. Spine.

[69] Emery CA, Rose MS, McAllister JR (2007). A prevention strategy to reduce the incidence of injury in high school basketball: a cluster randomized controlled trial. Clin J Sports Med.

[70] LaBella CR, Huxford MR, Grissom J (2011). Effect of neuromuscular warm-up on injuries in female soccer and basketball athletes in urban public high schools: cluster randomized controlled trial. Arch Pediatr Adolesc Med.

[71] Emery CA, Cassidy JD, Klassen TP (2005). Effectiveness of a home-based balance-training program in reducing sports-related injuries among healthy adolescents: a cluster randomized controlled trial. CMAJ.

[72] Barbic D, Pater J, Brison RJ (2005). Comparison of mouth guard designs and concussion prevention in contact sports: a multicenter randomized controlled trial. Clin J Sports Med.

[73] Holmich P, Larsen K, Krogsgaard K (2009). Exercise program for prevention of groin pain in football players: a cluster-randomized trial. Scand J Med Sci Sports.

[74] Hagglund M, Atroshi I, Wagner P (2013). Superior compliance with a neuromuscular training programme is associated with fewer ACL injuries and fewer acute knee injuries in female adolescent football players: secondary analysis of an RCT. Br J Sports Med.

[75] Krist MR, van Beijsterveldt AMC, Backx FJG (2013). Preventive exercises reduced injury-related costs among adult male amateur soccer players: a cluster-randomised trial. J Physiother.

[76] McIntosh AS, McCrory P, Finch CF (2009). Does padded headgear prevent head injury in rugby union football?. Med Sci Sports Exerc.

[77] Walden M, Atroshi I, Magnusson H (2012). Republished research: Prevention of acute knee injuries in adolescent female football players: cluster randomised controlled trial. Br J Sports Med.

[78] van Beijsterveldt AMC, van de Port IGL, Krist MR (2012). Effectiveness of an injury prevention programme for adult male amateur soccer players: a cluster-randomised controlled trial. Br J Sports Med.

[79] Soligard T, Myklebust G, Steffen K (2008). Comprehensive warm-up programme to prevent injuries in young female footballers: cluster randomised controlled trial. BMJ.

[80] Steffen K, Bakka HM, Myklebust G (2008). Performance aspects of an injury prevention program: a ten-week intervention in adolescent female football players. Scand J Med Sci Sports.

[81] Soligard T, Nilstad A, Steffen K (2010). Compliance with a comprehensive warm-up programme to prevent injuries in youth football. Br J Sports Med.

[82] Mattila VM, Sillanpaa PJ, Salo T (2011). Can orthotic insoles prevent lower limb overuse injuries? A randomized-controlled trial of 228 subjects. Scand J Med Sci Sports.

[83] Longo UG, Loppini M, Berton A (2012). The FIFA 11+ program is effective in preventing injuries in elite male basketball players: a cluster randomized controlled trial. Am J Sports Med.

[84] Steffen K, Meeuwisse WH, Romiti M (2013). Evaluation of how different implementation strategies of an injury prevention programme (FIFA 11+) impact team adherence and injury risk in Canadian female youth football players: a cluster-randomised trial. Br J Sports Med.

[85] Steffen K, Myklebust G, Olsen OE (2008). Preventing injuries in female youth football—a cluster-randomized controlled trial. Scand J Med Sci Sports.

[86] Larsen K, Weidich F, Leboeuf-Yde C (2002). Can custom-made biomechanic shoe orthoses prevent problems in the back and lower extremities? A randomized, controlled intervention trial of 146 military conscripts. J Manip Physiol Ther.

[87] Ronning R, Ronning I, Gerner T (2001). The efficacy of wrist protectors in preventing snowboarding injuries. Am J Sports Med.

[88] Pope RP, Herbert RD, Kirwan JD (2000). A randomized trial of preexercise stretching for prevention of lower-limb injury. Med Sci Sports Exerc.

[89] Withnall R, Eastaugh J, Freemantle N (2006). Do shock absorbing insoles in recruits undertaking high levels of physical activity reduce lower limb injury? A randomized controlled trial. J R Soc Med.

[90] Milgrom C, Finestone A, Lubovsky O (2005). A controlled randomized study of the effect of training with orthoses on the incidence of weight bearing induced back pain among infantry recruits. Spine.

[91] Lappe J, Cullen D, Haynatzki G (2008). Calcium and vitamin d supplementation decreases incidence of stress fractures in female navy recruits. J Bone Miner Res.

[92] Smith W, Walter JJ, Bailey M (1985). Effects of insoles in coast guard basic training footwear. J Am Podriatr Med Assoc.

[93] Gardner LI, Dziados JE, Jones BH (1988). Prevention of lower extremity stress fractures: a controlled trial of a shock absorbent insole. Am J Public Health.

[94] Schwellnus MP, Jordaan G, Noakes TD (1990). Prevention of common overuse injuries by the use of shock absorbing insoles. A prospective study. Am J Sports Med.

[95] Caraffa A, Cerulli G, Projetti M (1996). Prevention of anterior cruciate ligament injuries in soccer. Knee Surg Sports Traumatol Arthrosc.

[96] BenGal S, Lowe J, Mann G (1997). The role of the knee brace in the prevention of anterior knee pain syndrome. Am J Sports Med.

[97] Jorgensen U, Fredensborg T, Haraszuk JP (1998). Reduction of injuries in downhill skiing by use of an instructional ski-video: a prospective randomised intervention study. Knee Surg Sports Traumatol Arthrosc.

[98] Pope R, Herbert R, Kirwan J (1998). Effects of ankle dorsiflexion range and pre-exercise calf muscle stretching on injury risk in Army recruits. Aust J Physiother.

[99] Buchman AL, O’Brien W, Ou CN (1999). The effect of arginine or glycine supplementation on gastrointestinal function, muscle injury, serum amino acid concentrations and performance during a marathon run. Int J Sports Med.

[100] Holme E, Magnusson SP, Becher K (1999). The effect of supervised rehabilitation on strength, postural sway, position sense and re-injury risk after acute ankle ligament sprain. Scand J Med Sci Sports.

[101] Wedderkopp N, Kaltoft M, Lundgaard B (1999). Prevention of injuries in young female players in European team handball. A prospective intervention study. Scand J Med Sci Sports.

[102] Heidt RS, Sweeterman LM, Carlonas RL (2000). Avoidance of soccer injuries with preseason conditioning. Am J Sports Med.

[103] Torkki M, Malmivaara A, Reivonen N (2002). Individually fitted sports shoes for overuse injuries among newspaper carriers. Scand J Work Environ Health.

[104] Askling C, Karlsson J, Thorstensson A (2003). Hamstring injury occurrence in elite soccer players after preseason strength training with eccentric overload. Scand J Med Sci Sports.

[105] Perna FM, Antoni MH, Baum A (2003). Cognitive behavioral stress management effects on injury and illness among competitive athletes: a randomized clinical trial. Ann Behav Med.

[106] Kolt GS, Hume PA, Smith P (2004). Effects of a stress-management program on injury and stress of competitive gymnasts. Percept Mot Skills.

[107] Stasinopoulos D (2004). Comparison of three preventive methods in order to reduce the incidence of ankle inversion sprains among female volleyball players. Br J Sports Med.

[108] Van Tiggelen D, Witvrouw E, Roget P (2004). Effect of bracing on the prevention of anterior knee pain—a prospective randomized study. Knee Surg Sports Traumatol Arthrosc.

[109] Arnason A, Engebretsen L, Bahr R (2005). No effect of a video-based awareness program on the rate of soccer injuries. Am J Sports Med.

[110] Olsen OE, Myklebust G, Engebretsen L (2005). Exercises to prevent lower limb injuries in youth sports: cluster randomised controlled trial. Br Med J.

[111] Mohammadi F (2007). Comparison of 3 preventive methods to reduce the recurrence of ankle inversion sprains in male soccer players. Am J Sports Med.

[112] Collard DCM, Verhagen EALM, Chinapaw MJM (2010). Effectiveness of a school-based physical activity injury prevention program. Arch Pediatr Adolesc Med.

[113] Eils E, Schröter R, Schröder M (2010). Multistation proprioceptive exercise program prevents ankle injuries in basketball. Med Sci Sports Exerc.

[114] Knapik JJ, Trone DW, Swedler DI (2010). Injury reduction effectiveness of assigning running shoes based on plantar shape in Marine Corps basic training. Am J Sports Med.

[115] Bello M, Mesiano Maifrino LB, Gama EF (2011). Rhythmic stabilization versus conventional passive stretching to prevent injuries in indoor soccer athletes: a controlled clinical trial. J Bodyw Mov Ther.

[116] Franklyn-Miller A, Wilson C, Bilzon J (2011). Foot orthoses in the prevention of injury in initial military training: a randomized controlled trial. Am J Sports Med.

[117] Gomes EC, Allgrove JE, Florida-James G (2011). Effect of vitamin supplementation on lung injury and running performance in a hot, humid, and ozone-polluted environment. Scand J Med Sci Sports.

[118] Shih Y-F, Wen Y-K, Chen W-Y (2011). Application of wedged foot orthosis effectively reduces pain in runners with pronated foot: a randomized clinical study. Clin Rehabil.

[119] Hides JA, Stanton WR, Mendis MD (2012). Effect of motor control training on muscle size and football games missed from injury. Med Sci Sports Exerc.

[120] Cusimano MD, Chipman M, Donnelly P (2013). Effectiveness of an educational video on concussion knowledge in minor league hockey players: a cluster randomised controlled trial. Br J Sports Med.

[121] Askling CM, Tengvar M, Tarassova O (2014). Acute hamstring injuries in Swedish elite sprinters and jumpers: a prospective randomised controlled clinical trial comparing two rehabilitation protocols. Br J Sports Med.

[122] Drobnic F, Riera J, Appendino G (2014). Reduction of delayed onset muscle soreness by a novel curcumin delivery system (Meriva®): a randomised, placebo-controlled trial. J Int Soc Sports Nutr.

[123] Sebelien C, Maher S, Xianggui Q (2014). Effects of implementing Nordic hamstring exercises for semi-professional soccer players in Akershus, Norway. Orthop Pract.

[124] Sharma J, Weston M, Batterham AM (2014). Gait retraining and incidence of medial tibial stress syndrome in army recruits. Med Sci Sports Exerc.

[125] Childs JD, Teyhen DS, Casey PR (2010). Effects of traditional sit-up training versus core stabilization exercises on short-term musculoskeletal injuries in US Army soldiers: a cluster randomized trial. Phys Ther.

