# Supplementary material for: Compliance with Sport Injury Prevention Interventions in Randomised Controlled Trials: A Systematic Review
Source: Sports Med. 2016 Feb 11;46:1125–39. doi: 10.1007/s40279-016-0470-8 (PMC4963451; doi:10.1007/s40279-016-0470-8)
Supplement: Supplementary file 1 — Supplementary material 1 (DOCX 52 kb) [file 40279_2016_470_MOESM1_ESM.docx]

Electronic Supplementary Material Appendix S1**: INCLUDED STUDIES**

| **Section I** | | | | **Section II** | | **Section III** | | **Section IV** | | **Section V** |
| --- | --- | --- | --- | --- | --- | --- | --- | --- | --- | --- |
| **Authors [reference]** | **Year** | **Intervention** | **Furlan Score** | **Is compliance mentioned?** | **What is the term used?** | **Is compliance measured?** | **How is compliance measured?*** | **Are compliance rates provided?** | **Are compliance rates adjusted for?** | **What kind of analysis has been carried out? Intention-to-treat, or per-protocol-nalysis?** |
|  |  |  |  |  |  |  |  |  |  |  |
| Kraus et al. [52] | 1970 | Use of a helmet | 4 | YES | Use | YES | Visual check by supervisor | YES | NO |  |
| Ekstrand et al. [24] | 1983 | Prophylactic exercises and use of ankle tape | 1 | YES | Attendance | YES | Report by supervisor | NO | NO |  |
| Milgrom et al. [59] | 1985 | Use of orthotic insoles | 1 | YES | Use | YES | Visual check by supervisor | YES | NO |  |
| Smith et al. [98] | 1985 | Use of orthotic insoles | 1 | NO |  | NO |  | NO | NO |  |
| Gardner et al. [99] | 1988 | Use of orthotic insoles | 3 | YES | Compliance | NO |  | NO | NO |  |
| Schwellnus et al. [100] | 1990 | Use of neoprene insoles | 4 | YES | Compliance | YES | **Visual checks by researchers & self-report. Military study** | YES | NO |  |
| Sitler et al. [60] | 1990 | Use of a knee brace | 3 | YES | Compliance | YES | Visual check by researchers. Military study | YES | NO |  |
| Milgrom et al. [53] | 1992 | Use of modified shoes | 2 | YES | Use | YES | Visual check by supervisors. Military study | NO | NO |  |
| Schwellnus and Jordaan [48] | 1992 | Use of calcium supplements | 4 | YES | Compliance | YES | Report by supervisor. Military study | NO | NO |  |
| Barrett et al. [61] | 1993 | Use of designated shoes | 6 | YES | Use | YES | Visual check by supervisor | YES | NO |  |
| Van Mechelen et al. [33] | 1993 | Warm-up, cool-down and stretching exercises | 2 | YES | Compliance | YES | Self-report | YES | NO |  |
| Sitler et al. [62] | 1994 | Use of an ankle brace | 2 | YES | Compliance | YES | Visual check by researchers. Military study | YES | NO |  |
| Surve et al. [25] | 1994 | Use of ankle orthoses | 1 | YES | Compliance | YES | Report by supervisor | NO | NO |  |
| Caraffa et al. [101] | 1996 | Proprioceptive training | 2 | YES | Cooperation | NO |  | NO | NO |  |
| Bengal et al. [102] | 1997 | Use of a knee brace | 2 | NO |  | NO |  | NO | NO |  |
| Jorgensen et al. [103] | 1998 | Educational video | 3 | NO |  | NO |  | NO | NO |  |
| Pope et al. [104] | 1998 | Stretching exercises | 5 | NO |  | NO |  | NO | NO |  |
| Buchman et al.[105] | 1999 | Use of arginine supplements | 3 | NO |  | NO |  | NO | NO |  |
| Finestone et al. [54] | 1999 | Use of a designated shoe | 4 | YES | Compliance | YES | **Visual check by supervisors. Military study** | NO | NO |  |
| Holme et al. [106] | 1999 | Rehabilitation exercises | 3 | NO |  | NO |  | NO | NO |  |
| Wedderkopp et al. [107] | 1999 | Ankle disc training and warm-up exercises | 1 | NO |  | NO |  | NO | NO |  |
| Heidt et al.[108] | 2000 | Preconditioning program | 3 | NO |  | NO |  | NO | NO |  |
| Pope et al. [93] | 2000 | Stretching exercises | 4 | YES | Attendance | YES | **Visual check by supervisors and visits by researchers. Military study** | NO | NO | ITT |
| Soderman et al. [67] | 2000 | Balance board training | 1 | YES | Compliance and internal dropout | YES | **Self-report and report by supervisors** | YES | YES |  |
| Ronning et al. [91] | 2001 | Use of a wrist protector | 4 | NO |  | NO |  | NO | YES |  |
| Larsen et al. [88] | 2002 | Use of shoe orthoses | 7 | YES | Compliance | YES | Unknown | YES | YES | ITT & PPA |
| Larsen et al. [68] | 2002 | Strengthening exercises | 6 | YES | Compliance | YES | **Self-report and visual check by researchers** | NO | NO | ITT |
| Machold et al. [58] | 2002 | Use of an wrist protector | 6 | YES | Compliance | YES | Visual check by supervisors | YES | YES | ITT & PPA |
| Torkki et al. [109] | 2002 | Use of designated shoes | 5 | YES | Adherence | YES | Self-report | YES | NO |  |
| Asklin et al. [110] | 2003 | Preconditioning exercises | 3 | NO |  | NO |  | NO | NO |  |
| Knapik et al. [56] | 2003 | Physical readiness training | 3 | NO |  | YES | Visual check by supervisors. Military study | YES | NO |  |
| Perna et al. [111] | 2003 | Stress therapy | 5 | NO |  | NO |  | NO | NO |  |
| Wedderkopp et al. [34] | 2003 | Ankle disc training | 1 | YES | Compliance | YES | Self-report | NO | NO | ITT |
| Finestone et al. [112] | 2004 | Use of foot orthoses | 5 | YES | Use | YES | Visual check by supervisors. Military study | YES | NO |  |
| Kolt et al. [113] | 2004 | Stress therapy | 2 | NO |  | NO |  | NO | NO |  |
| Milgrom et al. [47] | 2004 | Use of risedronate supplement | 4 | YES | Compliance | YES | Visual check by supervisors. Military study | YES | NO | ITT & PPA |
| Sherry and Best [35] | 2004 | Rehabilitation exercises | 1 | YES | Compliance | YES | Self-report | YES | NO |  |
| Stasinopoulos [114] | 2004 | Proprioceptive exercises and external support | 2 | YES | Compliance | NO |  | NO | NO |  |
| van Tiggelen et al. [115] | 2004 | Use of a brace | 2 | NO |  | NO |  | NO | NO |  |
| Verhagen et al. [26] | 2004 | Balance board training | 4 | YES | Compliance | YES | Report by supervisor | NO | NO |  |
| Arnason et al. [116] | 2005 | Educational video | 3 | NO |  | NO |  | NO | NO |  |
| Barbic et al. [72] | 2005 | Use of a mouth guard | 4 | YES | Compliance | YES | **Report by supervisor and visits by researchers** | YES | NO | ITT |
| Emery et al. [71] | 2005 | Balance board training | 5 | YES | Compliance | YES | **Self-report and phone calls by researchers** | NO | YES | ITT |
| Finch et al. [63] | 2005 | Use of a mouth guard | 3 | YES | Compliance | YES | Visual check by supervisors | NO | NO |  |
| Milgrom et al. [96] | 2005 | Use of foot orthoses | 2 | YES | Compliance | YES | **Visual check by supervisors and visits by researchers. Military study** | NO | NO | ITT & PPA |
| Olsen et al. [117] | 2005 | Warm-up exercises | 7 | YES | Compliance | YES | Report by supervisor | YES | NO | ITT |
| Gabbe et al. [90] | 2006 | Preconditioning exercises | 4 | YES | Compliance | YES | Report by supervisor | YES | YES | ITT & PPA |
| Mcguine and Keene [28] | 2006 | Balance training | 5 | YES | Compliance | YES | Report by supervisor | NO | NO |  |
| Mickel et al. [64] | 2006 | Use of a brace and tape | 4 | YES | Compliance | YES | Visual check by supervisor | NO | NO |  |
| Withnall et al. [94] | 2006 | Use of insoles | 5 | NO |  | NO |  | NO | NO | ITT |
| Cobb et al. [43] | 2007 | Use of contraceptives | 5 | YES | Compliance | YES | Self-report | YES | YES | ITT & PPA |
| Emery et al. [69] | 2007 | Balance training | 6 | YES | Compliance | YES | **Self-report and report by supervisors.** | YES | NO |  |
| Hagglund et al. [29] | 2007 | Rehabilitation exercises | 5 | YES | Compliance | YES | Report by supervisor | YES | NO | ITT |
| Kekkonen et al. [44] | 2007 | Use of probiotics | 7 | YES | Adherence | YES | Self-report | YES | NO | ITT |
| Mohammadi [118] | 2007 | Proprioceptive & strength exercises plus use of orthoses | 2 | YES | Compliance | NO |  | NO | NO |  |
| Myklebust et al. [17] | 2007 | Balance training | 3 | YES | Compliance | YES | Report by supervisor | YES | YES | ITT |
| Noh et al. [36] | 2007 | Autogenic and relaxation training | 3 | YES | Adherence | YES | Self-report | YES | NO |  |
| Brushoj et al. [87] | 2008 | Strength, flexibility and coordination exercises | 6 | YES | Compliance | YES | Report by supervisor | YES | NO |  |
| Buist et al. [37] | 2008 | Graded training | 5 | YES | Compliance | YES | Self-report | YES | NO | ITT |
| Cumps et al. [38] | 2008 | Preventive exercises | 4 | YES | Compliance | YES | Self-report | YES | NO | ITT |
| Engebretsen et al. [31] | 2008 | Target exercises | 2 | YES | Compliance | YES | Self-report | YES | YES | ITT & PPA |
| Fredberg et al. [21] | 2008 | Prophylactic and stretching exercises | 1 | YES | Compliance | YES | Report by supervisor | YES | NO |  |
| Gilchrist et al. [19] | 2008 | Warm-up exercises | 4 | YES | Compliance | YES | Report by supervisor | YES | NO | PPA |
| Lappe et al. [97] | 2008 | Use of calcium and vitamin D supplements | 6 | YES | Use | YES | **Visual check and interview by researchers. Military study** | NO | NO | ITT & PPA |
| Pasanen et al. [16] | 2008 | Neuromuscular training | 6 | YES | Compliance and adherence | YES | Report by supervisor | YES | YES | ITT |
| Soligard et al. [79] | 2008 | Warm-up exercises | 5 | YES | Compliance | YES | **Report by supervisor; phone calls and visits by researchers** | YES | YES | ITT |
| Steffen et al. [85] | 2008 | Warm-up exercises (FIFA 11+) | 5 | YES | Compliance | YES | **Report by supervisor; phone calls and visits by researchers** | YES | YES | ITT |
| Holmich et al. [73] | 2009 | Preventive exercises | 4 | YES | Participation | YES | **Report by supervisor and visits by researchers** | NO | NO |  |
| Hupperets et al. [32] | 2009 | Proprioceptive exercises | 5 | YES | Compliance | YES | Self-report | YES | YES | ITT |
| Knapik et al. [55] | 2009 | Use of designated shoes | 3 | NO |  | YES | Visual check by supervisors. Military study | YES | NO |  |
| McIntosh et al. [76] | 2009 | Use of padded headgear | 4 | YES | Compliance | YES | **Report by supervisors and visits by researchers** | YES | YES | ITT |
| Childs et al. [132] | 2010 | Core stabilisation exercises | 5 | NO |  | NO |  | NO | NO |  |
| Collard et al. [119] | 2010 | Preventive exercises | 4 | NO |  | NO |  | NO | NO |  |
| Eils et al. [120] | 2010 | Proprioceptive exercises | 4 | NO |  | NO |  | NO | NO |  |
| Emery and Meeuwisse [66] | 2010 | Neuromuscular training | 3 | YES | Adherence | YES | **Self-report and report by supervisors** | YES | NO | ITT |
| Jamtveldt et al. [39] | 2010 | Stretching exercises | 5 | YES | Compliance | YES | Self-report | YES | NO | ITT |
| Kiani et al. [20] | 2010 | Motor skill exercises + education | 5 | YES | Compliance and adherence | YES | Report by supervisor | YES | YES |  |
| Knapik et al. [121] | 2010 | Use of customized shoes | 5 | NO |  | NO |  | NO | NO |  |
| Soligard et al. [81] | 2010 | Warm-up exercises | 5 | YES | Compliance | YES | **Report by supervisor; phone calls and visits by researchers** | YES | YES | ITT |
| Bello et al. [122] | 2011 | Stabilization exercises | 2 | NO |  | NO |  | NO | NO |  |
| Coppack et al. [22] | 2011 | Preventive exercises | 7 | YES | Compliance | YES | Report by supervisor | YES | NO |  |
| Franklyn-Miller et al. [123] | 2011 | Use of shoe orthoses | 5 | NO |  | NO |  | NO | NO |  |
| George et al. [65] | 2011 | Stabilization exercises | 6 | YES | Compliance | YES | Visual check by researchers. Military study | NO | NO |  |
| Gomes et al. [124] | 2011 | Vitamin C and E supplements | 6 | NO |  | NO |  | NO | NO |  |
| Kinchington et al. [49] | 2011 | Use of designated footwear | 4 | YES | Compliance | YES | Report by supervisor | YES | NO |  |
| Labella et al. [70] | 2011 | Neuromuscular training | 5 | YES | Compliance | YES | **Self-report and report by supervisors** | YES | NO |  |
| Mattila et al. [82] | 2011 | Use of foot orthoses | 9 | YES | Compliance | YES | **Self-report and visual check by researchers** | YES | NO | ITT |
| Mcguine et al. [50] | 2011 | Use of an ankle brace | 5 | YES | Compliance | YES | Report by supervisor | NO | NO | ITT |
| Parkkari et al. [40] | 2011 | Neuromuscular training | 7 | YES | Compliance | YES | Self-report | YES | NO | ITT |
| Petersen et al. [30] | 2011 | Eccentric training | 7 | YES | Compliance | YES | Report by supervisor | YES | NO |  |
| Ryan et al. [45] | 2011 | Use of designated shoes | 2 | YES | Use | YES | Self-report | NO | NO |  |
| Shih et al. [125] | 2011 | Use of foot orthoses | 5 | NO |  | NO |  | NO | NO |  |
| Beijsterveldt et al. [78] | 2012 | Preventive training (FIFA 11+) | 3 | YES | Compliance | YES | **Report by supervisors and visits by researchers** | YES | NO | ITT |
| Bredeweg et al. [95] | 2012 | Preconditioning program | 4 | YES | Compliance, adherence and exposure | YES | Self-report | YES | NO | ITT |
| Hides et al. [126] | 2012 | Motor control training | 4 | NO |  | NO |  | NO | NO |  |
| Longo et al. [83] | 2012 | Preventive training (FIFA 11+) | 7 | YES | Compliance | YES | **Report by supervisor; phone calls and emails by researchers** | YES | YES |  |
| Mcguine et al. [51] | 2012 | Use of ankle brace | 4 | YES | Compliance | YES | Report by supervisor | NO | NO |  |
| Walden et al. [77] | 2012 | Warm-up exercises | 6 | YES | Compliance | YES | **Report by supervisors and visits by researchers.** | YES | YES | ITT |
| Cusimano et al. [127] | 2013 | Educational video and brochure | 6 | NO |  | NO |  | NO | NO |  |
| Hagglund et al. [74] | 2013 | Neuromuscular training | 2 | YES | Compliance | YES | **Report by supervisors and visits by researchers** | YES | YES |  |
| Krist et al. [75] | 2013 | Preventive training (FIFA 11+) | 5 | YES | Compliance | YES | **Report by supervisors and visits by researchers** | YES | NO | ITT |
| Steffen et al. [84] | 2013 | Preventive training (FIFA 11+) | 2 | YES | Adherence | YES | **Report by supervisors and visits by researchers** | YES | YES | ITT |
| Steffen et al. [5] | 2013 | Preventive training (FIFA 11+) | 6 | YES | Adherence | YES | **Report by supervisors and visits by researchers** | YES | YES |  |
| Askling et al. [128] | 2014 | Rehabilitation exercises | 4 | NO |  | NO |  | NO | NO |  |
| Drobnic et al. [129] | 2014 | Use of curcumin | 8 | NO |  | NO |  | NO | NO |  |
| Janssen et al. [42] | 2014 | Neuromuscular training and use of an ankle brace | 3 | YES | Compliance | YES | Self-report | YES | NO | ITT |
| Sebelien et al. [130] | 2014 | Strengthening exercises | 5 | NO |  | NO |  | NO | NO |  |
| Sharma et al. [131] | 2014 | Gait retraining and flexibility exercises | 4 | NO |  | NO |  | NO | NO |  |
| Theisen et al. [46] | 2014 | Use of midsoles | 6 | YES | Use | YES | Self-report | YES | NO |  |

*Studies in bold provide double measures for compliance
